# Evaluation of the Yield of Histopathology in the Diagnosis of Lymph Node Tuberculosis in Morocco, 2017: Cross-Sectional Study

**DOI:** 10.2196/14252

**Published:** 2019-10-09

**Authors:** Kenza Bennani, Asmae Khattabi, Mohammed Akrim, Mohamed Mahtar, Najib Benmansour, Leila Essakalli Hossyni, Mehdi Karkouri, Nadia Cherradi, My Driss El Messaoudi, Ouafae Lahlou, Imad Cherkaoui, Yousef Khader, Abderrahmane Maaroufi, Salah-Eddine Ottmani

**Affiliations:** 1 Direction of Epidemiology and Diseases Control, Ministry of Health Rabat Morocco; 2 Morocco Field Epidemiology Training Program Rabat Morocco; 3 Ecole nationale de Santé publique Rabat Morocco; 4 Otorhinolaryngology Department, Hôpital du 20 Aôut Casablanca Morocco; 5 Otorhinolaryngology Department, Hôpital Hassan II Fez Morocco; 6 Otorhinolaryngology Department, Hôpital des Spécialités Rabat Morocco; 7 Histopathology Laboratory, Hôpital Ibn Rochd Casablanca Morocco; 8 Histopathology Laboratory, Hôpital des Spécilaités Rabat Morocco; 9 Tuberculosis Laboratory, Institut Pasteur Maroc Casablanca Morocco; 10 Tuberculosis National Reference Laboratory, Institut national d’Hygiène Rabat Morocco; 11 Department of Public Health, Jordan University of Science and Technology Amman Jordan; 12 Senior Expert on Tuberculosis Silves Portugal

**Keywords:** lymph node tuberculosis, yield, histopathology, positive predictive value, Morocco

## Abstract

**Background:**

The frequency of occurrence of extrapulmonary tuberculosis (EPTB) has been increasing globally over the last two decades. In Morocco, EPTB cases account for 46% of the patients reported with a new episode of tuberculosis (TB). Lymph node TB (LNTB) is the most common form of EPTB. In line with the guidelines of the National TB Program, the diagnosis is mainly based on clinical evidence, including histopathology.

**Objective:**

This study aimed to evaluate the yield of histopathology testing in the diagnosis of LNTB.

**Methods:**

This cross-sectional, prospective study was conducted among patients with cervical lymph node who were enrolled in the study from November 2016 to May 2017 in three regions of Morocco. We compared the outcomes of histopathological testing with those of bacteriology. Sensitivity (Se), specificity (Sp), positive predictive value (PPV), and negative predictive value (NPV) of histopathology testing were calculated. Culture and Xpert tests were used as the gold standard Laboratoty Testing.

**Results:**

A total of 262 patients were enrolled in this study. The Se, Sp, PPV, and NPV of histopathology testing were 95.6% (129/135), 64.6% (82/127), 74.1% (129/174), and 93.2% (82/88), respectively, in the presence of granuloma with or without caseous necrosis and were 84.4% (114/135), 74.8% (95/127), 78.1% (114/146), and 81.9% (95/116), respectively, in the presence of granuloma with caseous necrosis. The granuloma with caseous necrosis was associated with increased PPV and Sp of histopathology testing (P&lt;.05).

**Conclusions:**

The presence of the granuloma with caseous necrosis in the histopathological examination had significantly improved the yield of histopathology testing for the diagnosis of LNTB. The findings recommend to maintain histopathology testing in establishing the LNTB diagnosis and to explore other techniques to improve it.

## Introduction

Tuberculosis (TB) continues to challenge the international community. The global burden is estimated by the World Health Organization (WHO) at 10.4 million incident cases in 2016 [1]. The proportion of extrapulmonary TB (EPTB) cases has not exceeded 15% among all notified patients with TB for many years [1-3]. However, there are some differences across the WHO regions and across countries within these regions. The WHO Eastern Mediterranean Region (EMR) has the highest percentage of notified EPTB cases; 22% to 24% of patients diagnosed with TB each year have EPTB [1-3]. Among the EMR countries, Tunisia has the highest proportion of EPTB cases with 60% [3]. Significantly, high proportions are also reported in Algeria (63%), Syria (47%), Egypt (43%), and Saudi Arabia (26%) [1].

In Morocco, TB remains a major problem of public health. The WHO estimates for the year 2016 indicate that approximately 36,000 people were affected with a new episode of TB, with an estimated incidence of 103 per 100,000 population [1]. The National TB Program (NTP) of Morocco reported for the same year that 31,542 patients were diagnosed with a new episode of TB, and the notified incidence was 90 per 100,000 population [4]. The frequency of EPTB cases was high and accounted for 46% of all notified cases with a new episode of TB [1]. Between 1980 and 2016, the proportion of notified EPTB cases increased from 23% to 46% [5]. Lymph node TB (LNTB) is the most frequent form of EPTB and represents 37% of all EPTB cases reported in 2016 in Morocco.

According to the NTP guidelines of Morocco, the diagnosis of LNTB should be based on clinical arguments, including histopathological evidence. In fact, the diagnosis of 90% of notified LNTB cases is established through histopathology testing; bacteriological evidence contributes rarely to setting the diagnosis of LNTB [5]. Histopathology tests typically show epithelioid granulomas with or without caseous necrosis, which are usually used by clinicians as a strong argument to establish the diagnosis of TB. However, this histopathological aspect is not bacteriological evidence for TB and is encountered not only in TB but also in many other diseases such as sarcoidosis, leprosy, schistosomiasis, syphilis, and others.

The histopathology is highly sensitive but not very specific for TB diagnosis, in general. Therefore, using histopathology alone may result in a false diagnosis of EPTB. In addition, EPTB is characterized by a low presence of *Mycobacterium tuberculosis* (MBT) within the tissues because of an anaerobiosis environment. Therefore, the identification of MBT in biopsy fragments, through bacteriological techniques such as a culture or polymerase chain reaction (PCR), is not easy because of the paucibacillary nature of biopsies, and these examinations cannot provide optimal sensitivity [6]. This study aimed to evaluate the yield of histopathology testing for the diagnosis of LNTB in Morocco to help the NTP readjust the ongoing clinical procedures used to establish the diagnosis of LNTB.

## Methods

### Study Design

We conducted a cross-sectional, prospective study from November 2016 to May 2017 in otorhinolaryngology outpatient departments belonging to 4 university hospitals in 3 regions of Morocco (Rabat, Casablanca, and Fez). These regions have a high notified incidence of TB and appropriate laboratory facilities for histopathology and well-developed TB laboratory capacities. The study compares the outcomes of histopathological tests with those of bacteriological examinations using a culture and Xpert tests as the gold standard. The Xpert test was combined with the culture to strengthen the bacteriological gold standard, as its sensitivity (Se) and specificity (Sp), using lymph node biopsies, were, respectively, 96.6% and 88.9% [7].

### Sample

The study population consisted of all consenting patients with cervical lymph nodes, irrespective of their age and gender, for whom a biopsy was indicated to carry out histopathological testing to establish a diagnosis, including that of TB. All patients for whom the biopsy was contraindicated were excluded.

### Sample Size

In 2015, approximately 2000 new cases of LNTB were notified in 3 regions. Using an expected proportion of the bacteriological confirmation with histopathological characteristics suggesting TB of 68% [6], the sample size needed an alpha of .05, and power of 80% was estimated at 262 study participants.

### Laboratory Testing

Each lymph node biopsy carried out was examined by a histopathology test and by a culture on the Lowenstein-Jensen (LJ) solid medium and Xpert testing to provide the bacteriological evidence associated with TB. A bacteriological examination was considered positive when a culture or Xpert test or both were positive in a lymph node biopsy. A bacteriological examination was considered as negative when a culture and Xpert test were both negative in a lymph node biopsy. A histopathology test was considered positive when its outcome shows a presence of inflammatory granuloma with or without caseous necrosis. The presence of caseous necrosis in the granuloma was considered as suggestive of TB, whereas its absence as less suggestive, without ruling out the possibility of TB diagnosis. A histopathology test was considered negative when it shows no granuloma with or without nonspecific inflammatory lesions.

### Data Collection

The data to describe the population study were collected using a questionnaire filled by consenting patients, which included the demographic data (age, sex, and residence area [rural or urban area]), the socioeconomic data (education level, occupation/job, and income), and the clinical characteristics (previously treated for TB [having received TB medicines for ≥1 month], contacts of TB patients [household contacts or having close contact with TB patients], affected by diabetes, and HIV status). The results of histopathological and bacteriological tests were collected in the laboratories involved in the study using specific results sheets. All the staff involved in the process of data collection were trained to use the case definitions and collect the relevant information, in line with the study protocol, to standardize the data collection process.

Informed consent was obtained for each study participant, and all data obtained during the study were treated confidentially. The study was approved by the National Ethical Committee established by the Ministry of Health of Morocco.

### Data Analysis

The data were coded, verified, and analyzed using Epi Info version 7.2.1.0 (developed by Centers for Disease Control and Prevention in Atlanta, Georgia, US). We calculated the Se, Sp, positive predictive value (PPV), and negative predictive value (NPV) for each of the histopathological outcomes, namely (1) the granuloma with or without caseous necrosis and (2) the granuloma with caseous necrosis. To calculate these indicators, the outcomes of the histopathological tests were compared with the results of the gold standard (the culture and Xpert test). A chi-square test was used for the comparison of proportions. A chi-square test was considered statistically significant when the *P* value is less than .05.

## Results

### Sociodemographic and Clinical Characteristics

The study enrolled 262 patients with cervical lymph nodes from the following locations: 41.2% (108/262) in Casablanca, 35.1% (92/262) in Fez, and 23.7% (62/262) in Rabat. Their mean age was 25 years. Among the study participants, 151 were females (58.1%, 151/260), with a sex ratio (male/female) of 0.72. A total of 223 (87.4%, 223/255) patients were living in urban areas, 26 (10.2%, 26/255) were previously treated for TB, 6 (2.4%, 6/255) had diabetes, and 2 (1.7%, 2/115) were HIV positive (Table 1).

**Table 1:** Sociodemographic and clinical characteristics of study population.

Characteristics		Patients with lymph node (N=262), n (%)
**Age group (years; n=244)**		
	0-14	63 (25.8)
	15-44	150 (61.5)
	≥45	31 (12.7)
**Gender (n=260)**		
	Male	109 (41.9)
	Female	151 (58.1)
**Residency area (n=255)**		
	Urban	223 (87.4)
	Rural	32 (12.6)
**Education (n=231)**		
	None	49 (21.2)
	Coranic school	4 (1.7)
	Primary school	72 (31.2)
	Secondary school	75 (32.5)
	University	31 (13.4)
**Income (n=171)**		
	Stable	17 (9.9)
	Not regular	34 (19.9)
	No income	120 (70.2)
**Contact TB^a^** **patient (n=243)**		
	Yes	25 (10.3)
	No	218 (89.7)
**Previously treated for TB (n=255)**		
	Yes	26 (10.2)
	No	229 (89.8)
**Diabetes (n=255)**		
	Yes	6 (2.4)
	No	249 (97.6)
**HIV status (n=115**)		
	Positive rapid test	2 (1.7)
	Negative rapid test	113 (98.3)

**^a^**TB: tuberculosis.

### Outcomes of Histopathological and Bacteriological Examinations

Among the 262 enrolled patients, 174 (66.4%) had a positive histopathology test (granuloma with and without caseous necrosis), among whom 146 (83.9%, 146/174) had granuloma with caseous necrosis. Xpert testing was positive for 124 study participants (47.3%, 124/262) and culture, for 27 (10.3%, 27/262; Table 2).

**Table 2:** Histology, culture, and GeneXpert results.

Diagnostic technique		Patients with lymph nodes (N=262), n (%)
Positive histology		174 (66.4)
Granuloma with caseous necrosis		146 (83.9)
Granuloma without caseous necrosis		28 (16.1)
Negative histology		88 (33.6)
**GeneXpert**		
	Positive	124 (47.3)
	Negative	138 (52.7)
**Culture**		
	Positive	27 (10.3)
	Negative	235 (89.7)

### Histology Performances Compared With the Gold Standard

The Se, Sp, PPV, and NPV were 95.6% (129/135), 64.6% (82/127), 74.1% (129/174), and 93.2% (82/88), respectively, for the outcomes of histopathological tests, showing granuloma with or without caseous necrosis, whereas the Se, Sp, PPV, and NPV were 84.4% (114/135), 74.8% (95/127), 78.1% (114/146), and 81.9% (95/116), respectively, for the granuloma with caseous necrosis (Table 3). The PPV and Sp of histopathological outcomes were significantly higher for granuloma with caseous necrosis than for granuloma with or without caseous necrosis (*P*=.02 and *P*<.001, respectively).

**Table 3:** Performances of different histological entities in the diagnosis of lymph node tuberculosis.

Parameter	Histological entities				*P* value
	Granuloma with or without caseous necrosis, n/N (%)		Granuloma with caseous necrosis, n/N (%)		
	N	n (%)	N	n (%)	
Sensitivity	135	129 (95.6)	135	114 (84.4)	<.001
Specificity	127	82 (64.6)	127	95 (74.8)	.008
Positive predictive value	174	129 (74.1)	146	114 (78.1)	.02
Negative predictive value	88	82 (93.2)	116	95 (81.9)	.006

## Discussion

EPTB has much less visibility than pulmonary TB and receives less attention because of its low infectiousness [8,9]. However, it is a serious problem of public health because of its increasing frequency for the last 2 decades [1] and therefore contributing to morbidity, mortality, and disability associated with TB.

Significant variations in reporting notified EPTB cases have been observed among WHO regions and countries [1,3]. They might be related to differences in clinical practices to establish a diagnosis of EPTB, registration, and cases reporting.

In the context of Morocco, undertaking biopsy and histopathological testing is fully integrated in the clinical practices to establish the diagnosis of LNTB; the diagnosis of 90% of LNTB cases is based on histopathology evidence, whereas bacteriology testing is rarely used to confirm TB diagnosis [5] because of the very low yield of microscopy and time-consuming procedures to perform a culture on lymph node specimens; Xpert testing on such specimens has been recently introduced in the NTP services.

Our study has assessed the performances of histopathology against the established bacteriological gold standard. In general, the results of the study are comparable with those of other similar studies. In our study, the culture made on lymph node biopsies was positive in 10.3% (27/262) cases. According to the various studies, the culture positivity varied from 10% to 69%; it was 10.8% in Tunisia [10] and 45% in a study by Marais et al [11]. Other similar comparative studies had shown a positive culture of 22% [12], 26% [13], and 44% [14]. In these studies, a culture was performed on an LJ solid medium. Although a culture is the gold standard for the diagnosis of EPTB, a negative culture outcome cannot exclude the LNTB diagnosis [15].

Several studies reported Xpert testing on extrapulmonary biopsies with a positive outcome for TB of 11.5% [10], 33%, and 39% [10,14]. In our study, 47.3% (124/262) of Xpert tests performed on lymph node specimens were MTB positive.

The granuloma with caseous necrosis was observed in 83.9% (146/174) of the patients whose histopathology tests show a granuloma, irrespective of the caseous necrosis status. This proportion was more important than what was reported in the study by Lisbet et al (42.5%) [16]. Although a culture of MTB is generally used as a reference standard to validate the performance of new diagnostic tests, this test has a limited Se in extrapulmonary tissues [17]. In lymph node biopsies, the Se has been estimated at 71% to 88% whereas Sp, at 100% [18]. In our study, Xpert testing was used in addition to a culture to strengthen the bacteriological gold standard because its Se for EPTB ranged from 75% to 100% and its Sp, from 99% to 100% [19,20]. Its Se and Sp, using lymph node biopsies, were 96.6% and 88.9%, respectively [7]. Therefore, the gold standard based on a culture and Xpert testing may still have some limitations to evaluate the performance of histopathology in the diagnosis of LNTB.

With regard to the bacteriological gold standard used in our study, the outcomes of histopathology in favor of LNTB diagnosis were significantly better in the presence of granuloma with caseous necrosis than in the presence of granuloma with or without caseous necrosis.

Our study findings report that the Se, Sp, PPV, and NPV were 84.4% (114/135), 74.8% (95/127), 78.1% (114/146), and 81.9% (95/116), respectively, when the histopathological testing showed a granuloma with caseous necrosis, whereas these indicators were 95.6% (129/135), 66.4% (174/262), 74.1% (129/174), and 93.2% (82/88), respectively, when it showed a granuloma with or without caseous necrosis. These results were comparable with a study using a culture alone as the gold standard; this study carried out in Ethiopia reported an Se, an Sp, a PPV, and an NPV of 92%, 88%, 77%, and 97%, respectively, for LNTB when histopathology testing identifies a granuloma with or without caseous necrosis [21]. The Sp and PPV in our study were higher than those in another study using PCR as the gold standard with Se, Sp, PPV, and NPV estimated at 92%, 37%, 60%, and 81%, respectively [22]. The Se and Sp in our study were similar to those reported in another study (96% and 78%, respectively) [23].

Our study reported a 78% PPV in the presence of a granuloma with caseous necrosis; this indicates that 22% of patients might be considered as having LNTB whereas their culture and/or Xpert test are negative. The presence of caseous necrosis may represent a more comfortable argument for the diagnosis of LNTB than the situation when it is absent; however, it is still not compelling evidence that allows clinicians to set the diagnosis of LNTB with confidence because a significant proportion of the patients might not have TB (22% in our study).

The NPV exceeded 90% in the presence of granuloma with or without caseous necrosis and 83% in the presence of granuloma with caseous necrosis. Therefore, the proportion of false-negative cases would be 10% to 17%.

In conclusion, the presence of granuloma with caseous necrosis in a histopathological examination had significantly improved the yield of histopathology for the diagnosis of LNTB. The evaluation of clinical practices, including the histopathological examination in the diagnosis of LNTB in Morocco, indicates a better yield of histopathology when the histopathological testing shows a granuloma with caseous necrosis. Therefore, it is recommended to maintain the histopathology testing to establish the diagnosis of LNTB, explore other diagnosis techniques, and duplicate this study in other country settings.

## Abbreviations

EMR: Eastern Mediterranean Region
EPTB: extrapulmonary tuberculosis
LJ: Lowenstein-Jensen
LNTB: lymph node tuberculosis
MTB: mycobacterium tuberculosis
NPV: negative predictive value
NTP: National TB Program
PCR: polymerase chain reaction
PPV: positive predictive value
Se: sensitivity
Sp: specificity
TB: tuberculosis
WHO: World Health Organization
